# Host Adaptation and Speciation through Hybridization and Polyploidy in *Phytophthora*

**DOI:** 10.1371/journal.pone.0085385

**Published:** 2013-12-26

**Authors:** Lien Bertier, Leen Leus, Liesbet D’hondt, Arthur W. A. M. de Cock, Monica Höfte

**Affiliations:** 1 Department of Crop Protection, Ghent University, Ghent, Belgium; 2 Plant Sciences Unit, Applied Genetics and Breeding, Institute for Agricultural and Fisheries Research (ILVO), Melle, Belgium; 3 Fungal Biodiversity Centre (CBS-KNAW), Utrecht, The Netherlands; University of Arkansas, United States of America

## Abstract

It is becoming increasingly evident that interspecific hybridization is a common event in *phytophthora* evolution. Yet, the fundamental processes underlying interspecific hybridization and the consequences for its ecological fitness and distribution are not well understood. We studied hybridization events in *phytophthora* clade 8b. This is a cold-tolerant group of plant pathogenic oomycetes in which six host-specific species have been described that mostly attack winter-grown vegetables. Hybrid characterization was done by sequencing and cloning of two nuclear (ITS and *Ypt1*) and two mitochondrial loci (*Cox1* and *Nadh1*) combined with DNA content estimation using flow cytometry. Three different mtDNA haplotypes were recovered among the presumed hybrid isolates, dividing the hybrids into three types, with different parental species involved. In the nuclear genes, additivity, i.e. the presence of two alleles coming from different parents, was detected. Hybrid isolates showed large variations in DNA content, which was positively correlated with the additivity in nuclear loci, indicating allopolyploid hybridization followed by a process of diploidization. Moreover, indications of homeologous recombination were found in the hybrids by cloning ITS products. The hybrid isolates have been isolated from a range of hosts that have not been reported previously for clade 8b species, indicating that they have novel pathogenic potential. Next to this, DNA content measurements of the non-hybrid clade 8b species suggest that polyploidy is a common feature of this clade. We hypothesize that interspecific hybridization and polyploidy are two linked phenomena in *phytophthora*, and that these processes might play an important and ongoing role in the evolution of this genus.

## Introduction

*Phytophthora* is a genus of plant pathogenic filamentous oomycetes containing more than one hundred species. Virtually all of them are plant pathogens causing many important plant diseases worldwide, such as potato late blight, sudden oak death and forest dieback caused by *Phytophthora infestans*, *Phytophthora ramorum* and *Phytophthora cinnamomi*, respectively.

Morphologically, oomycetes are very similar to the filamentous fungi (kingdom Fungi). Therefore, they used to be classified as Fungi. Only during the last few decades, DNA and whole genome sequencing revealed that they have a completely different evolutionary origin but have adopted similar morphology and infection strategies through convergent evolution and horizontal gene transfer. Oomycetes differ from Fungi in some important morphological and biochemical aspects and in the fact that they are diploid in their vegetative lifestyle, whereas most Fungi are monoploid [1]. 

*Phytophthora* clade 8b contains a group of pathogens specifically adapted to cause disease at low temperatures in a range of important agricultural crops, mostly winter grown vegetables. A previous genetic diversity study of *Phytophthora* clade 8b isolates from around the world resulted in the official description of three new clade 8b species. This was based on differences in the nuclear rDNA internal transcribed spacer (ITS) and mtDNA cytochrome oxidase I (*Cox1*) barcoding sequences [2], morphological and physiological differences, and host preference [3]. The clade now consists of six host-specific species, namely *P. porri*, *P. primulae* and *P. brassicae* and the newly described *P. dauci*, *P. cichorii* and *P. lactucae*, respectively causing disease in leek, primrose, cabbages, carrot, chicory and lettuce. Two additional taxa were described, which have been tentatively named P. taxon castitis (on carrot and strawberry) and P. taxon parsley (on parsley), as insufficient data could be collected for an official species description. 

During this previous study [3], we detected intra-isolate sequence polymorphisms in the ITS region in 12 isolates, which were mainly isolated from *Allium* species. These sequence polymorphisms point to additivity, which is a distinctive feature of interspecific hybridization. Therefore, we decided to study the possible hybridity of these isolates using different techniques, which laid the foundation of this work. 

Natural interspecific hybridization has already been reported several times in the genus *Phytophthora* [4–12]. Next to this, synthetic hybrids have repeatedly been created in the lab [13–15]. In *Phytophthora*, the fundamental processes underlying interspecific hybridization and the consequences for its ecological fitness and distribution are not well understood. In plants, however, it is known that hybridization and polyploidy are important evolutionary processes contributing to adaptation and speciation and form the basis of the complete (angiosperm) biodiversity known today [16].

One of the main pathways of (angiosperm) speciation is believed to involve a polyploid phase, mostly occurring as a consequence of hybridization. In a hybridization event, (slightly) diverged chromosome sets are brought together in one nucleus. This causes unstable meiosis and in most cases abortion of the zygote. Fertility can be restored by a whole genome duplication event, creating again compatible chromosome sets. The resulting allopolyploid will then harbor the complete genomes of both parents. Although allopolyploids can be stable over long periods of time, it is well documented that most (allo)polyploids evolve back to a diploid state through the process of diploidization [17]. Diploidization can be defined as the transitioning of a polyploid organism back to the more stable diploid state. This can be accompanied by chromosome number reduction, although this is not necessarily the case. Processes that contribute to diploidization include subfunctionalization and/or neofunctionalization causing differentiation between redundant gene copies; large scale chromosomal rearrangements such as translocations; chromosome loss, etc. In allopolyploids, an important process that can create new, advantageous gene combinations and phenotypes is homeologous recombination, i.e. recombination between chromosomes of different parental origin [18]. 

Polyploidy has already been shown in several *Phytophthora* species [19–21]. In *P. infestans*, Sansome [20] showed that British isolates contained approximately twice the number of chromosomes compared to isolates from Mexico, the species’ center of origin. She hypothesized that *P. infestans* might exist in the tetraploid condition in temperate regions, and that the higher ploidy levels might enable the pathogen to adapt to cooler environments. This initiated a DNA content screening of *P. infestans* populations in many countries, using cytophotometric methods. Indeed, isolates from Mexico were found to contain much lower DNA contents compared to isolates from other regions [22], supporting Sansome’s hypothesis.

With the advent of the genomic era around the year 2000, research efforts aimed at understanding polyploidy in *Phytophthora* diminished. However, in 2010, a new study with recent *P. infestans* field isolates analyzed using flow cytometry showed large DNA content variation and heterokaryosis [23]. Moreover, by analysis of the genomes of *P. infestans*, *P. ramorum* and *P. sojae* using bio-informatics, remnants of an ancient polyploidization event were detected. Most likely, a common ancestor of these species has undergone a whole genome duplication that might have played a role in the evolution and pathogenic success of *Phytophthora* pathogens [24]. 

In this paper, we describe three different types of interspecific hybrids in *Phytophthora* clade 8b, as well as the occurrence of polyploidy as a common feature of the clade. We discuss a potential link between polyploidy and past hybridization events and the role that both events could play in host adaptation and speciation of *Phytophthora* pathogens. The implications of these phenomena for *Phytophthora* research are discussed.

## Materials and Methods

### Isolate collection and maintenance

All isolates used in this study are listed in Table 1. The isolates were freshly isolated from diseased plants or obtained from different culture collections around the world. Thirty-one of these isolates have been used previously in a genetic diversity study of *Phytophthora* clade 8b [3]. The isolates were maintained routinely on V8 agar [3] or on Corn Meal Agar (Beckton Dickinson). For long term storage, isolates were kept on V8 plugs at -80°C in 10% glycerol.

**Table 1:** Isolates used in this study.

**Species**	**Code** ^a^	**Alternative codes** ^b^	**Host**	**Origin**	**Year of isolation**	**Sequencing**			
						***Cox*I**	***Nadh*1**	**ITS**	**Ypt1**	
*Phytophthora porri*	CBS 802.95	PD 92/214	*Allium porrum*	Netherlands	1992	KC478717	-	KC478747	-	
	CBS 114100	-	*Allium porrum*	Denmark	1992	KC478718	KF882685	KC478748	KF882633	
	CBS 116662	Smilde GG	*Allium porrum*	UK	1994	KC478719	-	KC478749	-	
	CBS 127099	K06006(2)	*Allium porrum*	Belgium	2006	KC478720	KF882686	KC478750	KF882634	
	CBS 127101	S05029(1)	*Allium porrum*	Belgium	2005	KC478721	KF882687	KC478751	KF882635	
	CBS 181.87	-	*Allium porrum*	Netherlands	1987	-	-	FJ643565	-	
	CBS 127098	B06005(1)	*Allium porrum*	Belgium	2006	KF882609	KF882688	FJ643553	KF882636	
	S05014(1)	-	*Allium porrum*	Belgium	2005	KF882610	KF882689	FJ643551	KF882637	
	B06011	-	*Allium porrum*	Belgium	2006	KF882611	KF882690	-	KF882638	
	S05007(2)	-	*Allium porrum*	Belgium	2005	KF882612	KF882691	-	KF882639	
	S05017(2)	-	*Allium porrum*	Belgium	2005	KF882613	KF882692	-	KF882640	
	K05020(2)	-	*Allium porrum*	Belgium	2005	KF882614	KF882693	-	KF882641	
	K05025(1)	-	*Allium porrum*	Belgium	2005	-	-	-	-	
	B06008	-	*Allium porrum*	Belgium	2006	-	-	-	-	
	S05009	-	*Allium porrum*	Belgium	2005	-	-	-	-	
	K07015(4)	-	*Allium porrum*	Belgium	2007	-	-	-	-	
	S05012(2)	-	*Allium porrum*	Belgium	2005	-	-	-	-	
	K07015(2)	-	*Allium porrum*	Belgium	2007	-	-	-	-	
	S12001	-	*Allium porrum*	Belgium	2012	-	-	-	-	
*Phytophthora primulae*	CBS 110167	BBA 71108	*Primula eliator*	Germany	1999	KC478722	KF882694	KC478752	KF882642	
	CBS 116663	PD 99/2429	*Primula sp.*	Netherlands	1999	KC478723	KF882695	KC478753	KF882643	
	CBS 114346	LYN 916-A	*Primula polyantha*	New Zealand	2003	KC478724	KF882696	KC478754	KF882644	
	CBS 110162	BBA 70403	*Primula sp.*	Germany	1997	KC478725	KF882697	KC478755	KF882645	
	CBS 620.97	PD 97/875	*Primula acaulis*	Germany	1997	KC478726	KF882698	KC478756	KF882646	
	CBS 110165	-	*Primula sp.*	Germany	1998	KF882615	KF882699	KF882684	KF882647	
*Phytophthora* taxon parsley	BPIC 2584	-	*Petroselinum crispum*	Greece	2006	KC478727	KF882700	KC478757	KF882648	
	CBS 114156	-	*Petroselinum crispum*	Australia	2003	KC478728	KF882701	KC478758	KF882649	
*Phytophthora* taxon castitis	CBS 688.79	P3827	*Daucus carota*	Canada	1978	KC478729	KF882702	KC478759	KF882650	
	CBS 131246	CH112	*Fragaria x ananassa*	Sweden	1995	KC478730	KF882703	KC478760	KF882651	
*Phytophthora dauci*	CBS 127102	BorfSP370	*Daucus carota*	France	2009	KC478731	KF882704	KC478761	KF882652	
	CBS 114039	-	*Daucus carota*	Australia	2003	KC478732	KF882705	KC478762	KF882653	
*Phytophthora brassicae*	CBS 782.97	Smilde HH	*Brassica chinensis*	Netherlands	1994	KC478733	KF882706	KC478763	KF882654	
	CBS 212.82	P3273	*Brassica oleraceae*	Netherlands	1982	KC478734	KF882707	KC478764	KF882655	
	CBS 113350	PD 94/166	*Brassica oleraceae*	Netherlands	1994	KC478735	KF882708	KC478765	KF882656	
	CBS 112277	ICMP 14271	*Brassica oleraceae*	New Zealand	2001	KC478736	KF882709	KC478766	KF882657	
	CBS 127274	B10001	*Brassica oleraceae*	Belgium	2010	KC478737	KF882710	KC478767	KF882658	
	K13001	-	*Brassica oleraceae*	Belgium	2013	KF882616	KF882711	-	KF882659	
	CBS 179.87	-	*Brassica oleraceae*	Netherlands	1987	KF882617	AY564025	AF380148	KF882660	
	CBS 112967	-	*Brassica oleraceae*	UK	-	-	-	KF882682	-	
	CBS 686.95	-	*Brassica oleraceae*	Netherlands	1995	KF882618	KF882712	AF380149	KF882661	
	CBS 113352	-	*Brassica oleraceae*	Netherlands	1995	KF882619	KF882713	-	KF882662	
*Phytophthora lactucae*	BPIC 1985	-	*Lactuca sativa*	Greece	2001	KC478738	KF882714	KC478768	KF882663	
	BPIC 1988	-	*Lactuca sativa*	Greece	2002	KC478740	KF882715	KC478770	KF882664	
	BPIC 1992	-	*Lactuca sativa*	Greece	2003	KC478742	KF882716	KC478772	KF882665	
*Phytophthora cichorii*	CBS 115029	-	*Cichorium intybus*	Netherlands	2004	KC478743	KF882717	KC478773	KF882666	
	CBS 114345	-	*Cichorium intybus*	Netherlands	2003	KC478744	KF882718	KC478774	KF882667	
	CBS 115030	-	*Cichorium intybus*	Netherlands	2004	KC478745	KF882719	KC478775	KF882668	
	CBS 133815	SCRACE5388	*Cichorium intybus*	UK	1999	KC478746	KF882720	KC478776	KF882669	
Hybrid (type 1)	ICMP14653	-	*Allium cepa*	New Zealand	2002	KF882620	KF882721	KF882683	KF882670, KF882671	
	CBS 114040	-	*Pastinaca sativa*	Australia	-	KF882621	KF882722	Yes	KF882672	
	CBS 139.87	NBRC 30417, P6500	*Allium grayi*	Japan	1977	KF882622	KF882723	Yes	Yes	
	CBS 112966	-	*Chrysanthemum sp.*	UK	-	KF882623	KF882724	Yes	Yes	
	P6817	ADC 10.015	*Allium cepa*	Australia	1980	KF882624	KF882725	Yes	Yes	
	CBS 140.87	NBRC 30418, P7516	*Allium cepa*	Japan	1977	KF882625	KF882726	Yes	Yes	
	CBS 114101	P6815	*Parthenium argentatum*	Australia	1983	KF882626	KF882727	Yes	Yes	
	CBS 138.87	NBRC 30416, P6499	*Allium cepa*	Japan	1977	KF882627	KF882728	Yes	KF882673, KF882674	
	CBS 126739	MAFF237666, TAC 97-13, NBRC 32965	*Allium cepa*	Japan	1997	KF882628	KF882729	Yes	Yes	
	CBS 126738	MAFF 237665, TAC 97-5, NBRC 32964	*Allium victorialis*	Japan	1997	KF882629	KF882730	Yes	Yes	
	CBS 126737	MAFF 237664, TAC 97-1, NBRC 32963	*Allium victorialis*	Japan	1997	KF882630	KF882731	Yes	Yes	
Hybrid (type 2)	CBS 112969	ADC 03.38	*Allium cepa*	USA	-	KF882631	KF882732	Yes	KF882675, KF882676	
Hybrid (type 3)	CBS 112968	P6207	*Allium cepa*	Switzerland	-	KF882632	KF882733	Yes	KF882677, KF882678	
*Phytophthora syringae*	CBS 364.52	**-**	*Prunus armeniaca*	New Zealand	1952	HQ708406	AY564030	-	KF882679	
	CBS 114110	**-**	Almond	Australia	2004	HQ708407	KF882734	FJ643562	KF882680	
	CBS 110161	**-**	*Rhododendron*	Germany	1995	HQ708410	KF882735	FJ643561	KF882681	

^a^ All isolates starting with the letter codes CBS, BPIC, ICMP or P are publicly available at the CBS-KNAW Fungal Biodiversity center in The Netherlands, at the Benaki Phytopathological Institute Collection in Greece , at the International Collection of Microorganisms from Plants in New Zealand and at the World Phytophthora Collection at Riverside, California, respectively. All other isolates are stored in our private collection at the department of Crop Protection, Ghent University, but are available upon request.

^b^ Alternative codes are shown for easy comparison with other publications. ‘Yes’ means sequenced but not deposited to GenBank (due to polymorphisms caused by hybridization)

### DNA isolation, PCR, cloning and sequencing

*Phytophthora* isolates were grown in clarified V8 broth [3], for 7-10 days at 15°C in the dark. The mycelial mats were harvested by filtration, blotted dry, frozen in liquid nitrogen and pulverized using mortar and pestle. DNA was extracted using Qiagen’s DNeasy Plant Mini Kit (Hilden, Germany). The primers used in this study are shown in Table 2. PCR reactions for the nuclear ITS and *Ypt1* regions were performed in a 25 µL mix containing 2.5 µL 10x PCR buffer (Qiagen), 0.5 µL dNTPs (10 mM, Promega), 1 µL of each primer (10 µM), 0.15 µL Taq polymerase (5U/µL; Promega), 17.85 µL milli-Q water and 2 µL of DNA template (25 ng/µL). 

**Table 2:** List of primers used in this study.

**Locus**	**Primer (F or R)**	**Sequence (5’ – 3’)**	**Reference**
ITS	ITS1 (F)	TCC GTA GGT GAA CCT GCG G	[48]
	ITS4 (R)	TCC TCC GCT TAT TGA TAT GC	[48]
	ITSPA (F)	TTG TGG AGG CTG CCT GTA TG	This study
*Ypt1*	Ypt1F (F)	CGA CCA TYG GYG TKG ACT TT	[49]
	Ypt5R (R)	GCA GCT TGT TSA CGT TCT CR	[49]
*Cox1*	Oom-CoI-Lev-up (F)	TCA WCW MGA TGG CTT TTT TCA AC	[50]
	FM-85-mod (R)	RRH WAC KTG ACT DAT RAT ACC AAA	[50]
*Nadh1*	NADHF1 (F)	CTG TGG CTT ATT TTA CTT TAG	[51]
	NADHR1 (R)	CAG CAG TAT ACA AAA ACC AAC	[51]

For the mitochondrial genes (*Cox1* and *Nadh1*), the 25 µL reaction mixture contained 2.5 µL 10x PCR buffer (Qiagen), 3.5 µL MgCl_2_ (25 mM, Qiagen), 0.5 µL dNTPs (10 mM, Promega), 0.5 µL of each primer (10 µM), 0.15 µL Taq polymerase (5U/µL; Promega), 15.35 µL milli-Q water and 2 µL of DNA template (25 ng/µL). 

The amplifications were done in a Flexcycler PCR Thermal Cycler (Analytikjena). For ITS and *Ypt1* the following program was used: initial denaturation for 10 min at 94°C; 35 cycles of denaturation for 1 min at 94°C; annealing for 1 min at 60°C; extension for 1 min at 72°C; final extension for 10 min at 72°C. For the mtDNA genes, another program was used: initial denaturation for 10 min at 94°C; 40 cycles of denaturation for 1 min at 94°C; annealing for 30 sec at 52°C; extension for 1 min at 72°C; final extension for 10 min at 72°C. 

To reduce the impact of PCR mediated recombination [25] in the ITS region as was detected in our study, an improved PCR protocol was designed following the instructions suggested by Lahr and Katz [26]. More specifically, a new forward primer (ITSPA) was designed to reduce the size of the template to around 350 bp, now containing only the last 5 polymorphic base pairs between the P. *porri* and *P. primulae/P*. taxon parsley ITS sequences. Moreover, the amount of template was reduced to the absolute minimum needed for sufficient amplification and a proofreading polymerase (Phusion, NEB) was used. The reaction was performed in a 40 µL mix containing 8 µL Phusion HF buffer (5x, NEB), 0.8 µL dNTPs (10 mM, Promega), 1 µL of each primer (10 mM), 0.4 µL Phusion polymerase (20 U/mL, NEB), 27.8 µL milli-Q water and 2 µL of template (0.2 ng/µL). The thermal cycler was programmed as follows: initial denaturation for 30 sec at 98°C; 30 cycles of denaturation for 10 sec at 98°C; annealing for 30 sec at 55°C; extension for 20 sec at 72°C; final extension for 10 min at 72°C.

All PCR samples were held at 4°C before analysis by gel electrophoresis on 1% TAE gels containing Ethidium Bromide and evaluation under UV fluorescence. Before sequencing, PCR products were purified using the QiaQuick PCR Purification Kit (Qiagen). Both strands were sequenced by LGC Genomics (Berlin), using the same primers as for PCR. 

Cloning of ITS sequences was done using the CloneJET™ PCR Cloning Kit (Fermentas). Ten to twenty clones per isolate were sequenced for analysis. All newly generated sequence data have been submitted in GenBank (KF882609 - KF882735).

### Phylogenetic analysis

Alignments for the nuclear *Ypt1* and for the two mitochondrial (*Cox1* and *Nadh1*; appended) sequences, were made using MUSCLE (http://www.ebi.ac.uk/Tools/msa/muscle/), and manually edited afterwards using BioEdit. The two mtDNA genes represent about 4% of the mitochondrial genome, taking *P. ramorum* (clade 8c) as a reference for the mitochondrial genome size [27]. Phylogenetic analysis was done with the Maximum Likelihood algorithm using MEGA 5.2 [28]. Model testing was done using the software implemented in MEGA 5.2. For the mtDNA (*Cox1* and *Nadh1*) alignment, the HKY + G + I substitution model was chosen and for *Ypt1*, the K2 + I model was used. Bootstrapping was done with 1000 replicates. All alignments and phylogenetic trees are publicly available in Treebase (study number S14964; study url: http://purl.org/phylo/treebase/phylows/study/TB2:S14964)

#### DNA content analysis by flow cytometry

DNA contents were measured by flow cytometry for all isolates listed in Table 1, except CBS 112969. *Phytophthora* isolates were taken from storage at -80°C and grown in test tubes containing 5 mL clarified V8 broth for 6-10 days. The mycelium was then harvested and washed three times with sterile water. *Raphanus sativus* cv. Saxa was chosen as the internal DNA reference standard (with a genome size of 2C = 1.11 pg [29]). Nuclei extraction was done using the Cystain PI absolute P kit (Partec, Münster, Germany). For each sample approximately 0.5 cm^2^ of young *Raphanus* leaf tissue and a small amount of *Phytophthora* mycelium (around 1 mg of dry blotted mycelium) were co-chopped with a razor blade (Gilette) [30] in a Petri dish containing 500 µL extraction buffer. After chopping, the suspension was filtered through a 10 µm filter (CellTrics, Partec, Germany) and 2 mL of a Propidium Iodide staining solution was added. The samples were incubated overnight in the dark at 4°C. Measurements were done on a Partec PAS III flow cytometer (Partec, Germany) equipped with a 20 mW solid state laser (Sapphire 488-20) emitting at a fixed wavelength of 488 nm. The data were analyzed using Flomax software (Partec Münster, Germany). DNA content was calculated using the ratios between the peak positions of the *Phytophthora* sample and the *Raphanus* standard. The genome sizes are shown as pg DNA/2C in which 2C corresponds to the complete DNA content of the nucleus, irrespective of ploidy [31]. Histograms shown in this paper were made using Summit v4.3. The isolates were measured three times on different days over the course of three years. The coefficient of variation between the three repeated measures was on average 4.7%.

#### Oospore production and isolation

Oospore production was assessed for all hybrid isolates and for the parental species *P. porri*. Oospore production was induced by incubating a *Phytophthora* culture in a Petri dish containing V8 agar for one month at 15°C. Isolation of the oospores was done by digesting the mycelium using lysing enzymes from *Trichoderma harzianum* (Sigma). Ten mL of a solution containing 50 mg lysing enzymes was filter sterilized and pre-incubated overnight at 28°C. After this step, the solution was added to the Petri dish containing a *Phytophthora* culture and incubated for 2 days at 15°C. Hereafter, the solution containing oospores and mycelial fragments was scraped off the agar surface using a sterile spreader and filtered through a 70 µm filter (Cell Strainer, BD). Further removal of hyphal fragments was done by several rounds of centrifugation at low speed (1000 g), removing the supernatant and washing the pellet with sterile water. Oospores were visually examined under an Olympus BX51 microscope and the number of aborted oospores was counted. 

## Results

### Hybrid characterization

#### Polymorphic ITS sequences

The ITS region of 48 isolates (see Table 1) was amplified by primers ITS1 and ITS4 (Table 2) and sequenced. The ITS sequence of 12 isolates showed double peaks in the DNA sequence chromatogram, which indicates that these isolates have a putative hybrid origin. Nine of these isolates originated from diseased *Allium* spp., mostly onion (*Allium cepa*). The other three isolates were obtained from diseased *Parthenium argentatum*, *Chrysanthemum* sp. and *Pastinaca sativa*. The isolates mainly originated from Japan (6 isolates) and Australia (3 isolates). The other three isolates were found in the USA, UK and Switzerland (Table 1).

**Figure 1:**
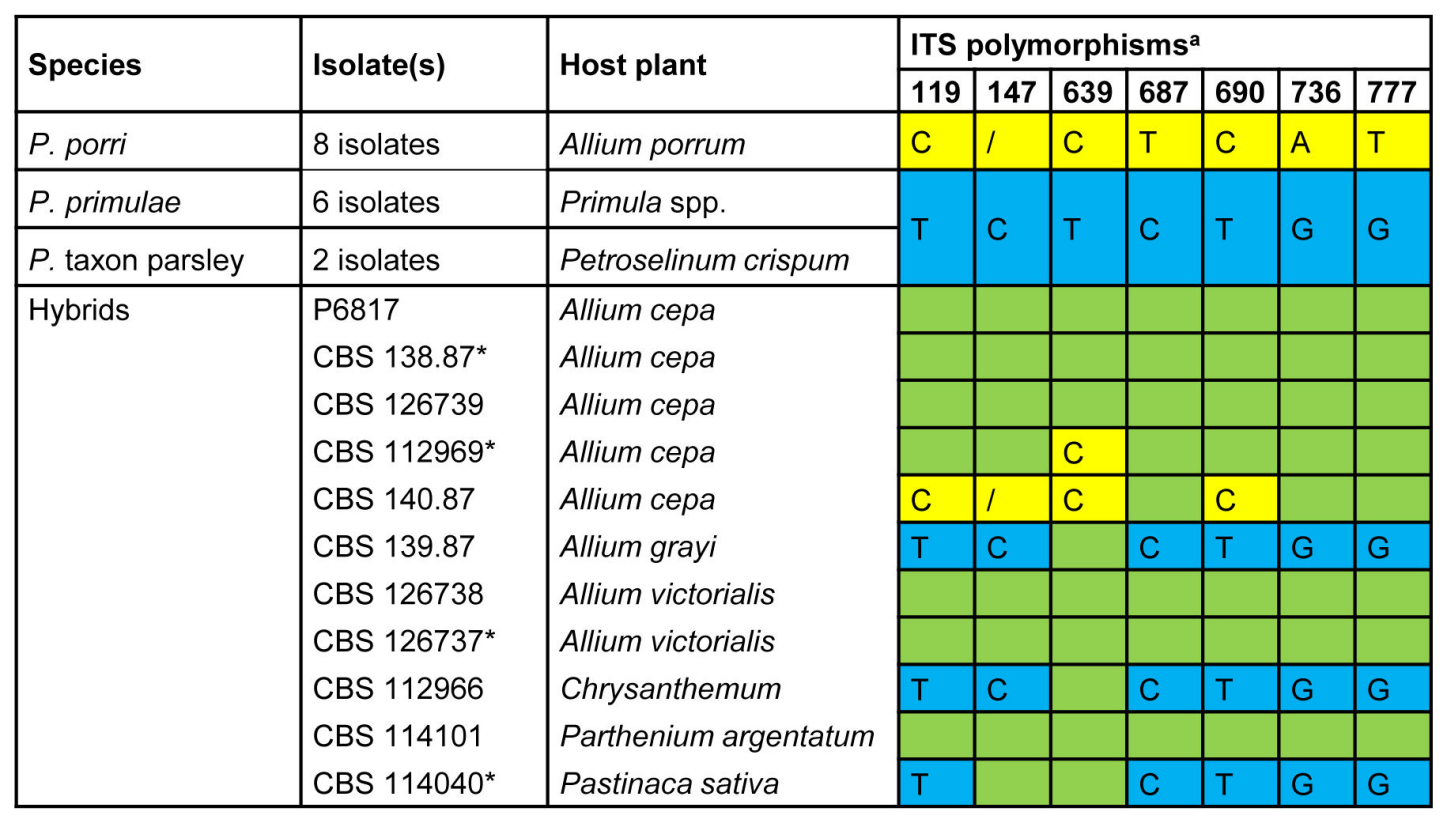
ITS polymorphisms for *P. porri*, *P. primulae*, *P*. taxon parsley and related hybrid isolates. ^a^ A green colour indicates a polymorphic position, i.e. that both parental states (blue and yellow) were present in the ITS amplicon * ITS cloning was done for these isolates: see Figure 2.

For 11 of these isolates, the polymorphic base pairs were found exactly at those positions (7 on a total of 806 bp) where the ITS sequence of *P. porri* differs from that of *P. primulae*/*P*. taxon parsley (with the latter two having identical ITS sequences). These 11 hybrid isolates are shown in Figure 1, along with the possible parental species *P. porri* and *P. primulae/P*. taxon parsley, which showed no intraspecific or intra-isolate variation in ITS. Six isolates were polymorphic (indicated with a green colour in Figure 1) at all seven distinctive base pairs. The other five isolates showed polymorphisms in only some of the distinctive base pairs (non-polymorphic sites are indicated with a blue or yellow colour in Figure 1). The non-polymorphic base pairs in a given isolate all corresponded to one of the two parental types, as if the ITS type is reverting back to one of the two parental states. 

The 12^th^ putative hybrid, isolate CBS 112968, showed intra-isolate polymorphisms at other positions in the ITS region and appeared not to be related to the other hybrid isolates. Instead, its ITS sequence was closely related to *P. cichorii*. 

#### Recombinant ITS types in the hybrids point to genetic recombination between the different parental genomes

**Figure 2:**
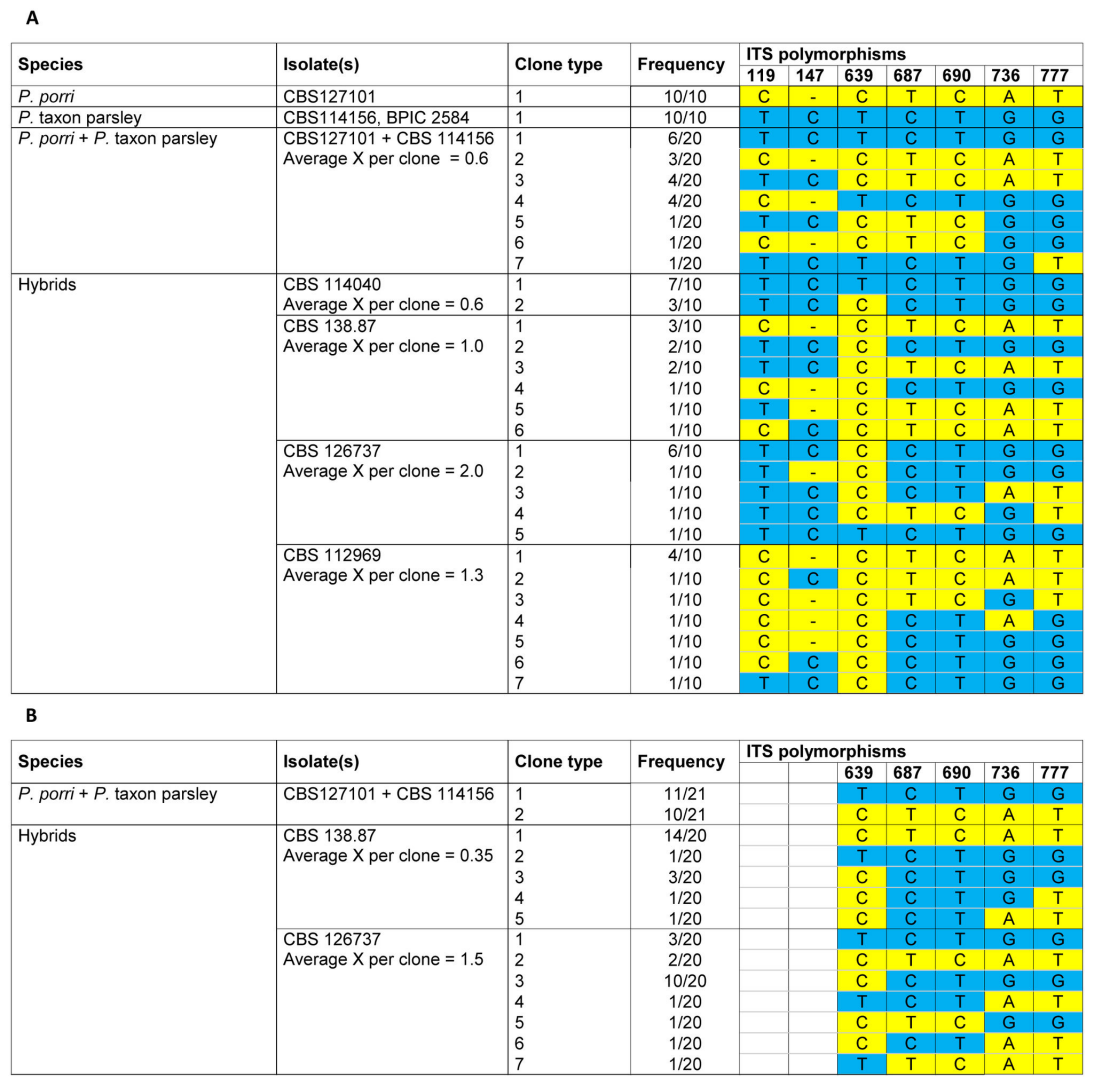
Cloning of ITS PCR products of hybrid isolates and their possible parental species. A: using Taq polymerase (with PCR artifact), B: using Phusion polymerase (without PCR artifact) X = recombination event.

By cloning and sequencing the polymorphic ITS amplification products, it is possible to identify the distinct haplotypes coming from the different parents. We cloned the ITS PCR product of four putative hybrids and analyzed the sequence of 10 clones per isolate (see Figure 2a). Interestingly, we found that most of the clones showed recombinant sequences between the P. *porri* and *P. primulae*/*P*. taxon parsley parental haplotypes, suggesting that genetic recombination between the different parental genomes has occurred. ITS products for the possible parental species *P. porri* and *P*. taxon parsley were also cloned. Here, all clones had identical sequences, aside from an occasional random SNP, which was never at the distinctive positions.

To assess the possible occurrence of recombination caused by a PCR artifact, which has been reported in studies on plant hybrids [25], a PCR reaction was run on the combined genomic DNA of *P. porri* and *P*. taxon parsley. From this experiment, it is clear that recombinant ITS clones can be formed due to a PCR artifact (Figure 2a). However, the average number of crossovers per clone (shown in Figure 2 as ‘average X per clone’) was always higher in the hybrids compared to the combined genomic DNA experiment.

To eliminate the bias caused by this PCR artifact, we optimized the PCR protocol following the instructions suggested by Lahr and Katz [26]. This protocol was again tested on a mixture of DNA of the two parental states (*P. porri* + *P*. taxon parsley) and twenty clones were sequenced (see Figure 2b). Here, no recombinant ITS clones were detected. In the hybrid isolates, however, again different types of recombinant ITS clones were found, leaving no doubt that this is a real biological phenomenon.

#### Sequencing of Ypt1 haplotypes to determine the parental origin of the hybrids

The ITS region is located in the rRNA genes that are repeated in tandem arrays of several hundreds of copies. Because of this organization, the ITS region is sensitive to concerted evolution, i.e. the homogenization of the DNA sequence of the different repeats, which makes this region less suitable to study the parental origin of the interspecific hybrids. For this reason, we decided to sequence the highly variable Ras-related *Ypt1* region [32], which is not sensitive to concerted evolution since it is a single copy gene.

**Figure 3:**
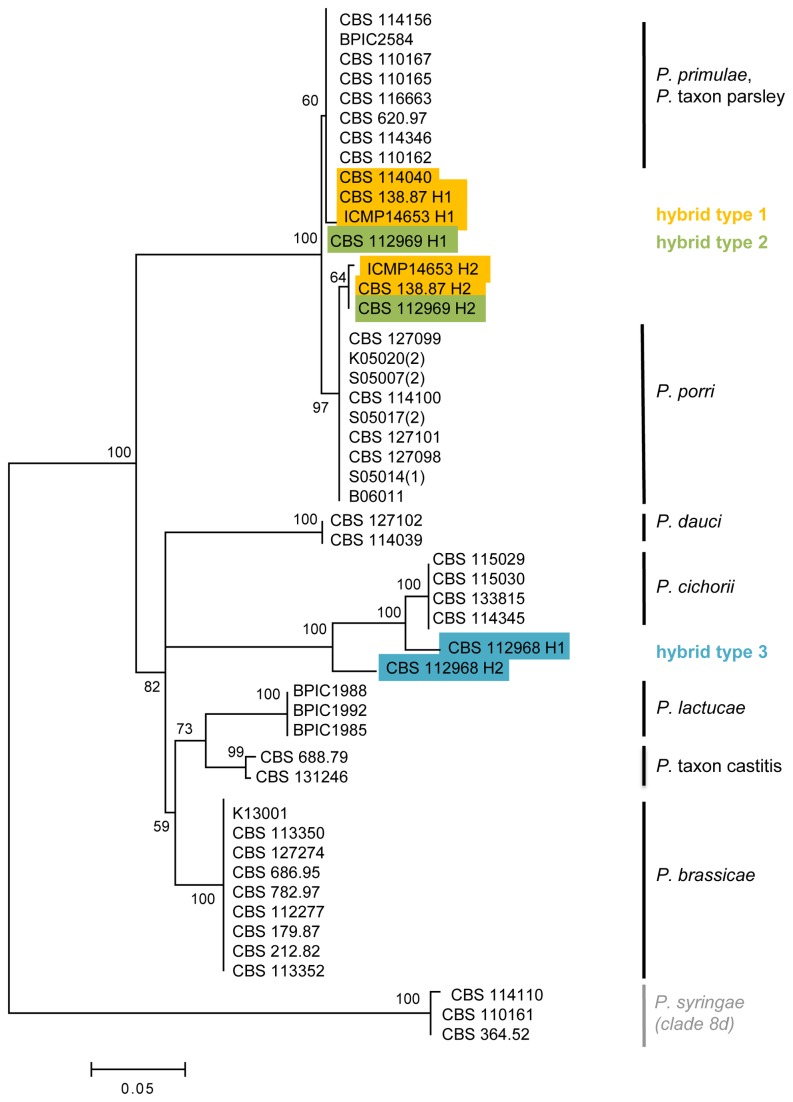
Ypt1 phylogeny of Phytophthora clade 8b. Phylogenetic tree derived from an alignment of Ypt1 sequence data of 42 clade 8b isolates. The Maximum Likelihood bootstrap support values are shown for all branches. The tree is rooted with three *P. syringae* isolates (clade 8d). For hybrid isolates CBS 138.87, ICMP14653 (hybrid type 1), CBS 112969 (hybrid type 2) and CBS 112968 (hybrid type 3) the different ITS haplotypes for the three hybrid types are shown in yellow, green and blue, respectively.

We sequenced the *Ypt1* region of 54 clade 8b isolates (see Table 1), including the hybrids. The resulting alignment length was 451 bp and a phylogenetic tree is shown in Figure 3. The interspecific variability in *Ypt1* sequence was much higher compared to ITS. Despite the high interspecific variability, the *Ypt1* locus showed almost no intraspecific variation and thus was very distinctive at the species level. The *Ypt1* sequences of *P. primulae* and *P*. taxon parsley, however, were identical. Most hybrid isolates showed polymorphic *Ypt1* sequences as observed by double patterns in the DNA sequence chromatogram. The only exception was isolate CBS 114040, which showed a sequence identical to that of *P. primulae/P*. taxon parsley. Remarkably, a polymorphic *Ypt1* sequence was also observed in isolate ICMP14653. This isolate was also collected from diseased onion, but had an ITS sequence identical to that of *P. primulae*/*P*. taxon parsley, without polymorphisms. The *Ypt1* gene was cloned for four hybrid isolates (CBS 138.87, ICMP14653, CBS 112968 and CBS 112969). For isolates ICMP14653, CBS 138.87 and CBS 112969, one haplotype was very similar to *P. primulae*/*P*. taxon parsley (0-2 SNPs) and the other haplotype was very similar to that of *P. porri* (2 SNPs). For isolate CBS 112968, two different haplotypes were recovered that were closely related but distinct from *P. cichorii*. The two distinct haplotypes for these isolates are shown in the phylogenetic tree in Figure 3. All other hybrid isolates showing a polymorphic *Ypt1* sequence are not shown in Figure 3.

#### mtDNA sequencing shows that at least three different hybridization events have occurred in clade 8b

**Figure 4:**
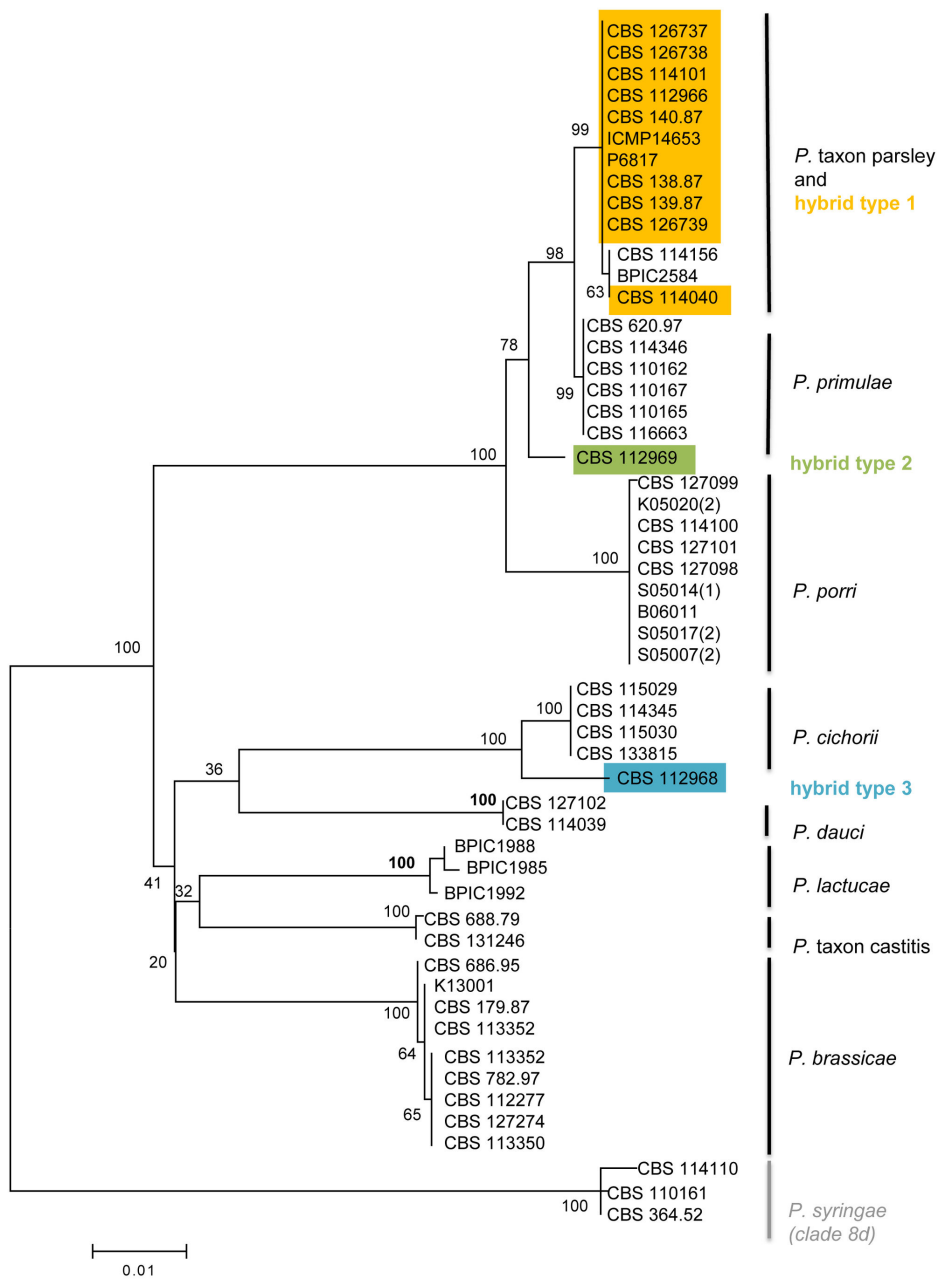
Multilocus mtDNA (Cox1 + *Nadh1*) phylogeny of Phytophthora clade 8b. Phylogenetic tree derived from a multilocus (appended) alignment of Cox1 and *Nadh1* sequence data of 50 clade 8b isolates. The Maximum Likelihood bootstrap support values are shown for all branches. The tree is rooted with three *P. syringae* isolates (clade 8d). Hybrid type 1 is shown in yellow, hybrid type 2 in green and hybrid type 3 in blue.

Since mitochondria are inherited maternally through the oogonia [33], sequencing of the mtDNA can identify the species that acted as the maternal parent in the interspecific cross. We sequenced two genes (*Cox1* and *Nadh1*) for 53 isolates and the appended alignment had a total length of 1539 bp. The multilocus phylogenetic tree is shown in Figure 4.

*Phytophthora* taxon parsley and most of the hybrid isolates (indicated as hybrid type 1 in Figure 4) form a cluster with high bootstrap support. Their closest relative was *P. primulae* which formed a separate cluster (99.7% sequence similarity, 5 SNPs). The hybrid isolate CBS 112969 (hybrid type 2 in Figure 4) had a different mtDNA sequence that showed most similarity to *P. primulae* (99.0% sequence similarity, 15 SNP’s), indicating that it was formed in a different hybridization event with an unknown species related to *P. primulae* acting as the maternal parent. The hybrid isolate CBS 112968 (hybrid type 3 in Figure 4), had a sequence most similar to that of *P. cichorii* (98.8 % sequence similarity, 18 SNP’s), demonstrating that this hybrid was formed in a third hybridization event with an unknown species related to *P. cichorii* acting as the maternal parent. 

#### DNA content measurements suggest polyploidy in different Phytophthora clade 8b species and are consistent with (allo)polyploidy in the hybrids

DNA content measurements were done for all isolates listed in Table 1 (except CBS 112969) and are presented in Table 3. Most *P. porri* isolates had a similar nuclear DNA content with a mean value of 0.270 pg DNA/2C (SE = 0.003, n = 14). One isolate (S05014(1)) contained approximately half of this DNA content (0.142 pg DNA/2C), suggesting polyploidy in this species, with the small DNA content most likely representing the diploid state. Moreover, four isolates contained a mixture of nuclei with two different DNA contents, with one nucleus type having a mean DNA content of 0.142 pg DNA/2C (SE = 0.002, n = 4) and the other having a mean DNA content of 0.273 pg DNA/2C (SE = 0.005, n = 4), suggesting that they are diploid/polyploid (probably diploid/tetraploid) heterokaryons. Histograms of the three different ploidy types are shown in Figure S1 (supporting data). 

**Table 3:** DNA content estimates of all *Phytophthora* clade 8b species and hybrids. *P. syringae* (clade 8d) is included for comparison .

**Species**	**Isolate(s)**	**Host plant**	**Mean (SE)** ^a^ **DNA content per nucleus (pg DNA/2C)**
*P. porri*	S05014(1)	*Allium porrum*	0.142
	B06011, S05007(2), S05017(2), K05020(2)		0.142 (0.002)/0.273 (0.005)
	CBS 181.87, CBS 802.95, CBS 114100, CBS 166662, CBS 127099, B06005(1), CBS 127101, K05025(1), B06008, S05009, K07015(4), S05012(2), K07015(2), S12001		0.270 (0.003)
*P. primulae*	CBS 620.97, CBS 110162, CBS 114346, CBS 110167, CBS 110165, CBS 116663	*Primula* spp.	0.269 (0.005)
*P*. taxon parsley	BPIC 2584	*Petroselinum crispum*	0.144/0.284
	CBS114156		0.171/0.347
Hybrid type 1	CBS114040	*Pastinaca sativa*	0.146
	CBS112966	*Chrysanthemum*	0.222
	CBS139.87	*Allium grayi*	0.223
	P6817	*Allium cepa*	0.237
	CBS140.87	*Allium cepa*	0.252
	ICMP14653	*Allium cepa*	0.276
	CBS114101	*Parthenium argentatum*	0.317
	CBS138.87	*Allium cepa*	0.326
	CBS126739	*Allium cepa*	0.337
	CBS126738	*Allium victorialis*	0.339
	CBS126737	*Allium victorialis*	0.388
*P. brassicae*	CBS113350	*Brassica* spp*.*	0.206
	CBS179.87, CBS782.97, CBS212.82, CBS686.95, CBS112277, CBS112967		0.277 (0.003)
	CBS113352, CBS127274, K13001		0.401 (0.010)
*P. dauci*	CBS127102, CBS114039	*Daucus carota*	0.269 (0.004)
*P. lactucae*	BPIC1985, BPIC1988, BPIC1992	*Lactuca sativa*	0.246 (0.002)
*P. cichorii*	CBS114345, CBS133815, CBS115029, CBS115030	*Cichorium intybus*	0.361 (0.011)
*P*. taxon castitis	CBS688.79, CH112	*Daucus carota, Fragaria x ananassa*	0.226 (0.010)
Hybrid type 3	CBS112698	*Allium cepa*	0.281
*P. syringae*	CBS114110, CBS110161, CBS36452	*Almond, Rhododendron, unknown*	0.176 (0.006)

^a^ SE is only shown where applicable, i.e. where more than one isolate was measured

For *P. primulae*, all isolates measured had a similar DNA content with a mean value of 0.269 pg DNA/2C (SE = 0.005, n = 6). The two P. taxon parsley isolates both contained two types of nuclei and are assumed to be diploid/polyploid (probably diploid/tetraploid) heterokaryons. One isolate (BPIC2584) had a DNA content comparable to the heterokaryotic *P. porri* isolates described above. The other isolate (CBS 114156) had a considerably higher DNA content.

The 11 hybrid type 1 isolates had highly variable DNA contents ranging from 0.146 to 0.388 pg DNA/2C. There is a positive correlation between the relative amount of polymorphic positions in the ITS PCR amplicon (coloured green in Figure 1) and the DNA content for a given hybrid (type 1) isolate (Pearson’s r, r = 0.690, n = 11, p = 0.019). This suggests that the hybrids evolve from a high DNA-content, allopolyploid state (such as in isolate CBS 126737) to a low DNA-content, diploid or diploid-like state (such as in isolate CBS 114040). 

Most *P. brassicae* isolates have a similar DNA content with a mean of 0.277 pg DNA/2C (SE = 0.003, n = 6), one isolate had a much lower DNA content (0.206 pg DNA/2C) and three isolates had a much larger DNA content of 0.401 pg DNA/2C (SE = 0.010, n = 2). For *P. cichorii*, the average DNA content was higher, with a mean value of 0.361 pg DNA/2C (SE = 0.011). The hybrid type 3 (isolate CBS 112968), had a DNA content of 0.281 pg DNA/2C. *Phytophthora lactucae* had a mean DNA content of 0.246 pg DNA/2C (SE = 0.002, n = 3), *P. dauci* had a mean DNA content of 0.269 pg DNA/2C (SE = 0.004, n = 2) and P. taxon castitis had a mean DNA content of 0.226 pg DNA/2C (SE = 0.010, n = 2).

For comparison we also measured the DNA content of *Phytophthora syringae*. This species belongs to clade 8d [34] and is one of the closest relatives to the clade 8b species. Its mean DNA content was 0.176 pg DNA/2C (SE = 0.006, n = 3). 

#### High levels of aborted oospores in the hybrids point to meiotic problems

Oospore production was assessed in ten hybrid type 1 isolates as well as for hybrid type 2 and 3 and for three isolates of the parental species *P. porri*. In most hybrid type 1 isolates, oospore production was highly impaired. Three of the hybrid type 1 isolates did not produce oospores (CBS 138.87, CBS 112966 and CBS 114040); three isolates produced them in extremely low amounts (P6817, CBS 140.87 and CBS 126739). The other four isolates (ICMP 14653, CBS 114101, CBS 126738 and CBS 126737) produced oospores in higher amounts.

**Figure 5:**
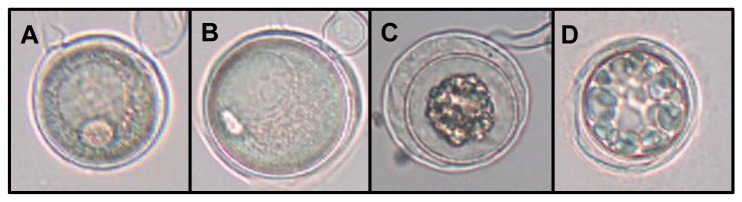
Normal and aborted oospores of hybrid type 1 (isolate CBS 126737). A,B: normal oospores; C,D: aborted oospores.

The number of abortive oospores was high in all six hybrid type 1 isolates that produced oospores (between 26.7% and 100%, see Table 4), and significantly higher than in isolates of *Phytophthora porri* (2.8% - 8.6%). In Figure 5, examples of normal and aborted oospores are shown. 

**Table 4:** Percentage of aborted oospores for the three hybrid types and for the parental species *P. porri*.

**Species**	**Isolate**	**Number of oospores counted**	**% of aborted oospores**
hybrid type 1	CBS 114040	13	100
	CBS 112966	0	N.A.
	CBS 10.015	0	N.A.
	CBS 140.87	34	82.4
	ICMP14653	138	77.5
	CBS 114101	108	74.1
	CBS 138.87	0	N.A.
	CBS 126739	9	66.7
	CBS 126738	173	61.3
	CBS 126737	330	26.7
hybrid type 2	CBS 112969	84	78.6
hybrid type 3	CBS 112968	139	8.6
*P. porri*	K11003	171	7.6
	CBS 127101	70	8.6
	S05010	142	2.8

Hybrid type 2 (CBS 112969) produced oospores, but a high amount was aborted (78.6%). Hybrid type 3 also produced oospores, but in this case only a low amount of aborted oospores was detected (8.6 %).

## Discussion

The increasing body of literature that is becoming available on hybridization in *Phytophthora* suggests that it could play an important role in the evolution of the genus. Natural interspecific hybridization events have been described in clade 1[5–7,9,11], clade 6 [10], clade 7 [4,12] and now in clade 8 (this study). From these previous studies, it is clear that interspecific hybridization has the potential to create new strains that have a new or expanded host range. The most striking example remains *Phytophthora alni*, which caused a new disease on alder trees across Europe and quickly became one of the most serious threats in riparian ecosystems. The pathogen was proven to be of recent hybrid origin and contains an evolving group of allopolyploid genotypes [35].

The clade 8b hybrids were genetically characterized by cloning and sequencing two nuclear regions (ITS and *Ypt1*) and sequencing of two mtDNA genes (*Cox1* and *Nadh1*). Hereby, three different types of hybrids could be distinguished with different parental species involved in their formation. The largest group (hybrid type 1, 11 isolates) originated from hybridization between *P. porri* and *P*. taxon parsley. Hybrid type 2 (isolate CBS 112969) was closely related to the hybrid type 1, but has a different maternal parent which is an unknown species related to *P. primulae*. Hybrid type 3 (isolate CBS 112968) is unrelated to the first two types, and was formed in a hybridization event between two unknown species closely related to *P. cichorii*. There is a clear discrepancy between the nuclear (*Ypt1*) and mitochondrial (*Cox1* + *Nadh1*) phylogenies. While most clade 8b species show a much faster mutation rate in the *Ypt1* gene (as visualized by longer branch lengths) compared to the mtDNA genes, the reverse is true for *P. porri* and *P. primulae*. This demonstrates that gene flow (hybridization events) between *P. porri* and *P. primulae* is common in evolutionary terms. Moreover, hybrid types 1 and 2 both have mtDNA haplotypes different from *P. primulae* and *P. porri*, while their *Ypt1* haplotypes are nearly identical to those of *P. porri* and *P. primulae*. This inconsistency is probably due to backcrossing or other reticulation events.

We have measured DNA contents for over 50 isolates belonging to the different *Phytophthora* clade 8b species and for the hybrids. In *P. porri*, where 18 isolates were measured, we detected intraspecific DNA ploidy variation. The majority of the isolates were polyploid (probably tetraploid), one isolate was diploid, and four isolates were diploid/polyploid heterokaryons. Previous research in our lab [36] has shown that isolates belonging to these three different ploidy types, have identical AFLP patterns. In the case of allopolyploid hybrids, AFLP patterns would differ significantly from the parental patterns (i.e. a combination of AFLP fragments from both parents) [5]. Therefore, the data suggest that in *P. porri*, a whole genome duplication has occurred, creating an autopolyploid. 

In *P. primulae*, no intraspecific DNA content variation was detected and the mean DNA content was similar to that of the polyploid *P. porri* isolates, suggesting that also *P. primulae* is polyploid. Both isolates of P. taxon parsley were diploid/polyploid heterokaryons. In *P. brassicae*, three different DNA contents were found in the nine isolates that were analyzed. Most likely, these DNA content variations also represent ploidy variations. In the other clade 8b species, no considerable intraspecific DNA content variation was detected, but DNA contents were always in the polyploid range.

*P. syringae* (clade 8d), one of the closest relatives to the clade 8b species, had a DNA content of 0.176 pg DNA/2C. Isolates of *P. ramorum* (clade 8c) have also been evaluated for DNA content by flow cytometry using the same protocol as was used in our study [37]. These isolates had a stable DNA content of 0.146 pg DNA/2C (SE = 0.002, n = 3), which is assumed to represent the diploid state. 

The hybrid type 1 isolates show a large variation in DNA content. This variation in DNA content was positively correlated with additivity (i.e. the presence of two alleles) in the nuclear loci. The isolates with the highest DNA content showed complete additivity in both nuclear loci, which is what is to be expected from a stable or recently formed allopolyploid. The isolates with the smallest DNA contents show almost no additivity in ITS, and in one isolate (CBS 114040) only one *Ypt1* haplotype is still present. Processes that can cause this loss of additivity include chromosome loss, non-reciprocal translocations, gene conversion and unequal crossing over [18,38].

Recombinant ITS sequences between the two parental types were detected by cloning and sequencing of different hybrid isolates. Theoretically, these recombinations can be due to either asexual, mitotic recombination, or sexual recombination in the form of homeologous recombination. Asexual or mitotic recombination is in general a rare event that is associated with double strand break repair [39]. Since the hybrid isolates also exhibit problems in meiosis, as shown by high numbers of aborted oospores, homeologous recombination is likely to have occurred. Although it is unclear to what extent homeologous recombination occurs in other parts of the genome, it can be an important process of creating novel genetic variation as has been described for plant hybrids [18].

Our study raised some concern on the use of ITS as the universal barcode for identification of *Phytophthora*, since hybridization events can be easily overlooked. The ITS region is located in the rRNA genes that are repeated in tandem arrays of several hundreds of copies. Because of this organization, the ITS region is sensitive to concerted evolution, i.e. the homogenization of the DNA sequence of the different repeats. From plant research, it is known that the ITS can evolve in three different ways after interspecific hybridization: (1) unidirectional concerted evolution leads to loss of one of the parental copies and fixation of the other (2) formation of a new ITS type that represents a mixture of the two parental types, (3) presence of both ITS types, which is the case in young hybrid taxa, although this situation can also be stable in older hybrid taxa [40–42]. In the clade 8b hybrids, unidirectional concerted evolution is ongoing, keeping one of the two parental haplotypes and in this way erasing the footprints of hybridization.

Most *Phytophthora* species show a certain degree of host specificity, and this is also the case for the clade 8b species. Hence, the question remains how and where different species can meet intimately, a requirement for interspecific hybridization to occur. Since the clade 8b species show a clear host preference for winter grown field vegetables that are commonly used in crop rotation systems (such as leek, carrot, cabbages, lettuce, parsley, chicory), it is likely that the different species can be present on the same field. Moreover, all three hybrid types, formed in hybridization events with different parental species, have been isolated from onion (*Allium cepa*). This led us to hypothesize that *Allium cepa* might be a common susceptible host of the different clade 8b species involved in the hybrids’ formation, offering a hot spot for interspecific hybridization to occur. Pathogenicity tests with *P. porri*, one of the two parents of hybrid type 1 and 2, show that it can easily infect onion (unpublished results). Pathogenicity tests with the other clade 8b species on onion, could shed more light on this hypothesis.

Of the hybrid type 1, six isolates were derived from *Allium cepa*, the other isolates were derived from *Allium victorialis*, *Allium grayi*, *Pastinaca sativa*, *Chrysanthemum* sp. and *Parthenium argentatum*, indicating a clear expansion of host range compared to the parents that were so far only detected on leek (*P. porri*) and parsley (*P*. taxon parsley).

As a result of globalization, plants and therefore their pathogens are now being traded all over the world on a large scale. This evidently increases the chance of interspecific hybridization since closely related species or different genotypes of the same species that have been geographically isolated are brought together while their reproductive barriers might be incomplete [43].

Therefore, the implications of hybridization on the adaptive potential and pathogenicity of *Phytophthora* spp. deserve to be studied in more detail. An outstanding topic of discussion in this respect is the fate of genes involved in plant pathogen interaction. In the past few decades, it has become clear that *Phytophthora* pathogens are extremely flexible and this has made it a very challenging task for plant breeders to develop crops with durable resistance against *Phytophthora*. A lot of attention has been given to the biology of the RXLR effectors that have been discovered recently [44,45]. These effector genes have been coined as one of the major determinants of host specificity. From whole genome sequencing, we know that a single pathogenic *Phytophthora* isolate can contain high numbers of different and often unique RXLR effector genes (563 different RXLR effector genes predicted from the P. *infestans* genome sequence [45]). It has also been discovered that the genome of *P. infestans* (with its 250 Mb the largest oomycete and chromalveolate genome sequenced up till now) consists for 74% of repetitive sequence, contributing to the flexibility of the genome. 

It is easy to imagine how (allopolyploid) hybridization and its consequences could have helped to build up the enormous effector repertoires that we find in *Phytophthora* pathogens today. The fact that effector genes are mainly contained in gene sparse regions in the DNA enables recombination between effector gene regions by homeologous recombination, without high risks of lethality. 

Intra-specific hybridization between divergent races of the same species could also enhance the pathogen’s pathogenic potential by expansion and/or recombination of effector gene repertoires. In *P. ramorum*, a heterothallic species, crosses have been induced between different genotypes that have diverged as a consequence of geographical isolation [46]. The resulting oospore progenies were characterized by DNA content estimation and microsatellite genotyping [37]. A large proportion of the progeny showed DNA contents that were considerably larger (up to 70%) than their parental DNA content. Microsatellite patterns also indicated polyploidy and aneuploidy in the progeny.

The same processes might be responsible for the increased pathogenic potential observed for *P. infestans* in Europe since the 1980s, when sexual reproduction of the pathogen was enabled by the introduction of the A2 mating type from its center of origin to Europe. In a recent study [47], herbarium samples of the P. *infestans* strains that caused the Irish potato famine in the 19^th^ century were collected and their genomes were sequenced. These genomes were compared to the genomes of recent aggressive *P. infestans* strains that have outcompeted the old strains. An important difference between the old strains (that were dominant for about 50 years) and the new strains, is the rise in ploidy level. The old strains were found to be diploid, while the new strains are mostly triploid and sometimes tetraploid, which was estimated based on allele frequency from the resequencing alignments. It is unclear what caused this rise in ploidy level, but in our opinion, hybridization between divergent *P. infestans* genotypes, seems to be a likely scenario.

In conclusion, we have found that interspecific hybridization has occurred different times in clade 8b with different parental species involved. The combination of sequencing different genes and estimating DNA content by flow cytometry provided valuable insights in the processes involved in and after interspecific hybridization. A recently formed hybrid will contain the complete complement of both parental genomes (allopolyploidy). Novel gene combinations such as a combined effector repertoire can enable the pathogen to invade new habitats or hosts. After allopolyploidization, different genetic processes such as chromosome loss and chromosomal rearrangements (which can be between the different parental genomes through homeologous recombination), can enable the pathogen to further adapt to a new habitat or host. As can be seen in the hybrid type 1 isolates, the progeny of a single hybridization event can follow different evolutionary paths, which ultimately can result in the formation of new species. It is clear from our data that the footprints of hybridization can easily be lost. Therefore, it is possible that the occurrence and importance of hybridization in *Phytophthora* is highly underestimated. We hypothesize that the occurrence of DNA content variation and polyploidy in *Phytophthora* species is linked with (ancient) hybridization events. 

## Supporting Information

Figure S1 **Ploidy variation in *P. porri*.** DNA content measurements on mycelial nuclei of three different *P. porri* isolates using the internal standard *Raphanus sativa* cv. Saxa (2C = 1.11 pg). A, logarithmic histogram with ‘diploid’ *P. porri* (isolate S05014(1)); B, histogram with ‘tetraploid’ *P. porri* (isolate CBS 127098); C, histogram with heterokaryotic ‘diploid/tetraploid’ *P. porri* (isolate S05007(2)); Pp1: *P. porri* G1 peak; Pp2, *P. porri* G2 peak (panels A and B) or second G1 peak (panel C); Pp3: *P. porri* G2 peak; Rs1: *Raphanus sativa* G1 peak; Rs2: *Raphanus sativa* G2 peak.
(PPTX)
