# Supplementary material for: Host Adaptation and Speciation through Hybridization and Polyploidy in Phytophthora
Source: PLoS One. 2013 Dec 26;8(12):e85385. doi: 10.1371/journal.pone.0085385 (PMC3873470; doi:10.1371/journal.pone.0085385)
Supplement: Figure S1 — Ploidy variation in P. porri. DNA content measurements on mycelial nuclei of three different P. porri isolates using the internal standard Raphanus sativa cv. Saxa (2C = 1.11 pg). A, logarithmic histogram with ‘diploid’ P. porri (isolate S05014(1)); B, histogram with ‘tetraploid’ P. porri (isolate CBS 127098); C, histogram with heterokaryotic ‘diploid/tetraploid’ P. porri (isolate S05007(2)); Pp1: P. porri G1 peak; Pp2, P. porri G2 peak (panels A and B) or second G1 peak (panel C); Pp3: P. porri G2 peak; Rs1: Raphanus sativa G1 peak; Rs2: Raphanus sativa G2 peak. (PPTX) [file pone.0085385.s001.pptx]

## Slide 1
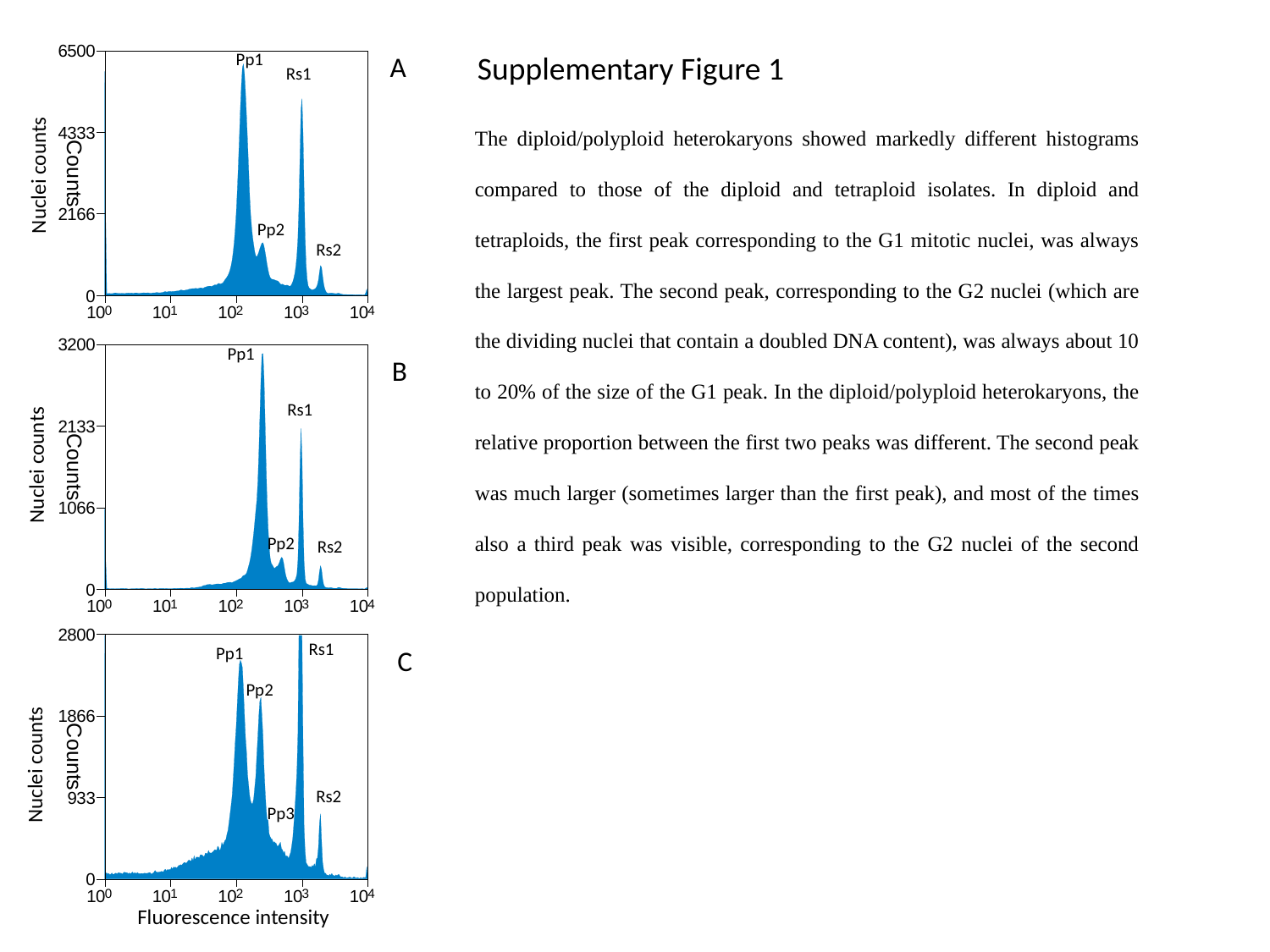

Pp1
A
Rs1
Nuclei counts
Pp2
Rs2
Pp1
B
Rs1
Nuclei counts
Pp2
Rs2
Rs1
Pp1
C
Pp2
Nuclei counts
Rs2
Pp3
Fluorescence intensity
Supplementary Figure 1
The diploid/polyploid heterokaryons showed markedly different histograms compared to those of the diploid and tetraploid isolates. In diploid and tetraploids, the first peak corresponding to the G1 mitotic nuclei, was always the largest peak. The second peak, corresponding to the G2 nuclei (which are the dividing nuclei that contain a doubled DNA content), was always about 10 to 20% of the size of the G1 peak. In the diploid/polyploid heterokaryons, the relative proportion between the first two peaks was different. The second peak was much larger (sometimes larger than the first peak), and most of the times also a third peak was visible, corresponding to the G2 nuclei of the second population.
